# From maternal microbial transmission to neonatal gut microbiota restoration: the potential and challenges of maternal fecal microbiota transplantation

**DOI:** 10.3389/fmicb.2026.1972665

**Published:** 2026-10-29

**Authors:** Xia Hu, Guangyu Ma, Jinbiao Han, Lijuan Zhu, Ying He, Dan Zhu

**Affiliations:** 1 Department of Gynecological Nursing, West China Second University Hospital, Sichuan University, Chengdu, China; 2 Key Laboratory of Birth Defects and Related Diseases of Women and Children, Ministry of Education, West China Second University Hospital, Sichuan University, Chengdu, China; 3 Department of Nursing, Meishan Women and Children’s Hospital, Meishan, Sichuan, China; 4 Reproductive Medical Center, Department of Obstetrics and Gynecology, West China Second University Hospital, Sichuan University, Chengdu, China; 5 Department of Obstetrics and Gynecology, West China Second University Hospital, Sichuan University, Chengdu, China; 6 Department of Emergency Medicine, Meishan Women and Children’s Hospital, Meishan, Sichuan, China; 7 Health Management Center, Meishan Women and Children’s Hospital, Meishan, Sichuan, China

**Keywords:** early-life microbiota, fecal microbiota transplantation, maternal microbial transmission, microbial engraftment, microbiota restoration, neonatal gut microbiota, vertical transmission

## Abstract

Early-life gut microbiota establishment is a critical developmental process shaped by maternal microbial transmission and ecological succession. Microorganisms from the maternal gut, vagina, breast milk, skin, and oral cavity contribute to neonatal microbial acquisition, while strain-resolved studies increasingly support the maternal gut as an important reservoir of strains capable of persistent colonization in the infant intestine. This transmission can be disrupted by cesarean delivery, prematurity, perinatal antibiotic exposure, and neonatal intensive care, resulting in delayed acquisition of maternal commensals, altered microbial succession, and increased colonization by opportunistic microorganisms. Current restoration strategies, including probiotics, synbiotics, and vaginal microbiota seeding, can modify early microbial communities but do not fully reproduce the diversity, maternal specificity, and persistence of naturally transmitted maternal gut strains. Maternal fecal microbiota transplantation (FMT) has therefore emerged as a potential strategy to restore disrupted maternal gut-to-infant microbial transmission. Initial proof-of-concept and strain-resolved studies suggest that carefully screened maternal FMT can increase mother-to-infant strain sharing, promote recovery from cesarean-associated microbiota perturbations, and reduce colonization by potential pathogens. However, evidence for durable engraftment, functional benefits, clinical efficacy, and long-term safety remains limited. Future studies should prioritize rigorous donor screening, standardized intervention protocols, appropriate recipient selection, and longitudinal integration of strain-resolved metagenomics, metabolomics, immune profiling, and clinical outcomes. Maternal FMT remains investigational but provides a promising framework for precision early-life microbiota restoration.

## 1 Introduction

Early-life gut microbiota establishment represents a critical developmental window with profound implications for immune maturation, metabolic programming, and neurodevelopmental trajectories across the lifespan (Rautava et al., 2012; Tamburini et al., 2016; Jian et al., 2021). Rather than being a purely stochastic process, neonatal gut microbiota development is increasingly recognized as a maternally influenced ecological process shaped by microbial transmission during the perinatal period. Maternal microbial communities from the gut, vagina, breast milk, oral cavity, and skin collectively provide important sources of microorganisms for the developing infant microbiota (York, 2018; Ma et al., 2023, 2024; Xiao and Zhao, 2023). Through maternal-to-infant transmission and subsequent ecological selection and succession, these microorganisms contribute to the assembly and maturation of the neonatal gut microbiota and the developing host–microbiota relationship.

Contemporary perinatal conditions and clinical practices, including cesarean delivery, preterm birth, perinatal antibiotic exposure, neonatal intensive care, and formula feeding, can disrupt this process (Drell et al., 2014; Bokulich et al., 2016; Gregory et al., 2016; Shao et al., 2019; Morreale et al., 2023; Xie et al., 2025b). Such perturbations may alter the timing and sources of microbial acquisition, reduce the transmission of key maternal commensals, and redirect normal microbiota succession during a critical developmental period. These effects may extend beyond microbial composition itself by influencing the maturation of host–microbiota interactions and early-life physiological development.

Several strategies have therefore been explored to restore disturbed early-life microbiota development. Probiotics can introduce selected beneficial microorganisms, but their effects are strain specific and they cannot reproduce the diversity and ecological interactions of naturally transmitted maternal microbial communities (Tannock, 2021; He et al., 2024). Vaginal microbiota seeding has similarly been proposed to restore birth-associated microbial exposure in cesarean-delivered infants, yet its clinical benefits remain uncertain, and concerns persist regarding safety, interindividual variability, and the relevance of vaginal microorganisms to persistent infant gut colonization (Mueller et al., 2019; Jenmalm, 2020; Wang et al., 2024). In contrast, accumulating strain-resolved evidence indicates that the maternal gut is a major reservoir of microorganisms capable of persistent colonization in the infant intestine. This has shifted attention from supplementation of individual taxa toward restoration of maternal gut-derived microbial transmission.

Within this framework, maternal FMT has emerged as an investigational strategy for modifying early-life gut microbiota development by introducing a complex, maternally derived gut microbial community during neonatal colonization. Unlike supplementation with individual strains, maternal FMT may facilitate the transfer of diverse maternal gut-derived microorganisms. However, greater similarity to the microbiota of vaginally delivered infants or increased maternal–infant strain sharing does not, in itself, demonstrate improved health. Vaginally delivered infants provide a useful reference for characterizing delivery-associated microbial differences, but their microbiota should not be assumed to represent a universal optimal state. The clinical rationale for microbiota restoration therefore depends on whether the targeted microbial differences are associated with impaired microbial function or adverse health outcomes. Clinical translation also requires careful consideration of donor screening, pathogen transmission, microbial engraftment, optimal timing and dosage, long-term safety, and appropriate recipient selection.

Previous reviews have examined maternal microbial transfer and microbiota-based interventions for cesarean-associated infant dysbiosis (Liu et al., 2024; Jurgiel et al., 2026). Building on these reviews, we critically evaluate maternal FMT by distinguishing microbial transfer and engraftment from functional changes and clinical benefits. We also compare its rationale, evidence, and safety considerations with those of alternative microbiota-targeted interventions and identify key gaps that must be addressed before clinical application.

## 2 Maternal microbial transmission and neonatal gut microbiota establishment

Neonatal gut microbiota establishment is a dynamic ecological process shaped by maternal, dietary, host, and environmental factors (Stewart et al., 2018; Ma et al., 2023). Among these, maternal-to-infant microbial transmission represents a major route of early microbial acquisition, with microorganisms from the maternal gut, vagina, breast milk, skin, and oral cavity contributing through distinct transmission pathways (Figure 1) (Ferretti et al., 2018; Valles-Colomer et al., 2023). However, microbial exposure alone does not determine successful colonization. Following transmission, ecological selection, strain-specific fitness, microbial interactions, and temporal succession determine which microorganisms establish and persist in the neonatal gut.

**Figure 1:**
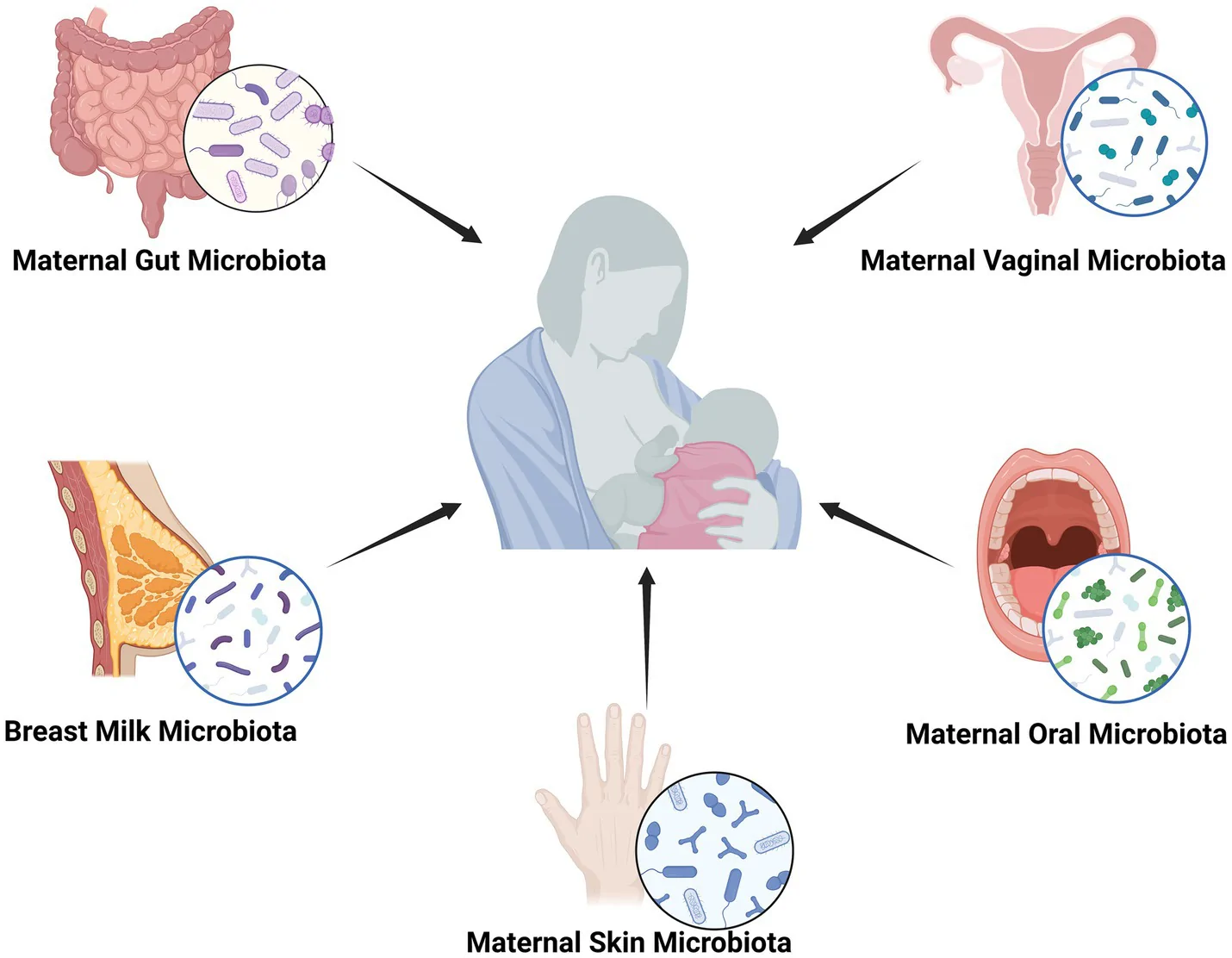
Maternal sources of microorganisms transmitted to the infant. Maternal microorganisms originate from multiple body sites, including the gut, vagina, breast milk, skin, and oral cavity, and are transmitted to the infant through distinct perinatal and postnatal pathways. These maternal microbial sources collectively contribute to early microbial exposure and subsequent establishment of the infant microbiota.

### 2.1 Maternal gut microbiota

The maternal gut microbiota represents a major reservoir of microorganisms for the developing infant gut and is among the maternal microbial sources with the greatest potential for persistent colonization. With the advent of strain-resolved metagenomics, mother-to-infant microbial sharing has been shown to extend beyond the genus or species level to highly similar or identical bacterial strains (Asnicar et al., 2017; Ferretti et al., 2018; Yassour et al., 2018). These studies provided direct evidence that the maternal intestine is an important source of early-life gut colonizers.

Longitudinal metagenomic analyses across multiple maternal body sites further demonstrated that infants rapidly acquire microorganisms from the maternal gut, vagina, and skin after birth, but that strains from different maternal sources differ markedly in their persistence within the infant intestine. Among these sources, maternal gut-derived strains contribute most substantially and persist more effectively than strains derived from other maternal body sites (Ferretti et al., 2018). Subsequent work similarly showed that infant strain persistence is strongly associated with maternal origin and strain-specific ecological characteristics, further supporting the central role of maternal gut-derived strains in long-term infant colonization (Lou et al., 2021).

This concept has recently been reinforced by a large longitudinal metagenomic study of 714 mother–infant pairs and 4,526 fecal samples. The maternal gut microbiome was identified as the predominant reservoir of infant gut strains, whereas transmission from the maternal vaginal and breast milk microbiota occurred only occasionally. Mother–infant gut strain sharing was strongly time dependent, and a higher maternal abundance of a given species increased the likelihood of its transmission. Moreover, maternally shared strains were more likely to persist throughout infancy (Sinha et al., 2026). Transmitted strains were enriched in genes related to carbohydrate transport and metabolism, coenzyme transport and metabolism, and potential immunomodulatory functions, suggesting that successful transmission and persistence may depend partly on functional adaptation to the early-life intestinal environment.

Such selective transmission is particularly relevant to early-life commensals such as *Bifidobacterium* and *Bacteroides*, whose successful establishment depends on strain availability and the capacity to adapt to the infant intestinal environment. Together, these findings identify the maternal gut as an important reservoir of strains contributing to early-life gut community assembly and provide a biological basis for investigating maternal gut-derived microbiota restoration.

### 2.2 Vaginal microbiota transmission during delivery

Vaginal delivery represents one of the most direct opportunities for neonatal exposure to maternal microorganisms. During passage through the birth canal, newborns are extensively exposed to microorganisms from the maternal vagina and perineal region. Early community-level studies showed that the microbiota of vaginally delivered neonates more closely resembled the maternal vaginal microbiota, whereas cesarean-delivered neonates showed greater representation of skin-associated microorganisms, suggesting that delivery mode strongly influences the earliest microbial exposures after birth (Dominguez-Bello et al., 2010).

Subsequent longitudinal and strain-resolved studies, however, have emphasized the distinction between microbial exposure during delivery and persistent colonization of the infant gut. Maternal vaginal strains may be detected in infants shortly after birth but generally show lower persistence in the infant intestine than maternal gut-derived strains (Ferretti et al., 2018). Consistently, analyses of maternal vaginal and infant stool microbiomes have demonstrated limited transmission of vaginal strains to the neonatal gut even after vaginal delivery, indicating that passage through the birth canal alone does not determine persistent intestinal colonization (Dos Santos et al., 2023).

Recent large-scale metagenomic evidence further supports this distinction. The maternal gut, rather than the vaginal microbiome, appears to be the predominant reservoir of infant gut strains, although vaginally carried gut-adapted species may occasionally establish successfully. For example, in all five families in which dominant maternal vaginal *Bifidobacterium breve* strains could be reconstructed, the same strains were detected in the corresponding infant guts (Sinha et al., 2026). This observation suggests that ecological compatibility with the intestinal niche may be more important for long-term persistence than the magnitude of exposure alone.

Importantly, the influence of vaginal delivery on infant gut microbiota development should not be reduced to direct “seeding” by typical vaginal microorganisms. Comparisons of vaginal, planned cesarean, and emergency cesarean deliveries suggest that delivery mode influences early microbial stability and colonization patterns through multiple interconnected mechanisms (Mitchell et al., 2020). These may include differences in exposure to maternal gut and perineal microorganisms, perinatal antibiotics, and healthcare environments. Cesarean delivery has been associated with altered microbiota development during the first year of life (Reyman et al., 2019), reduced transmission of maternal *Bacteroides* strains, and increased colonization by hospital-associated opportunistic bacteria (Shao et al., 2019). Thus, the microbiological significance of vaginal delivery likely extends beyond exposure to vaginal taxa and instead reflects preservation of a broader maternal microbial transmission process that facilitates acquisition of gut-adapted maternal strains.

### 2.3 Breast milk microbiota and breastfeeding

Maternal-to-infant microbial transmission continues after delivery, and breastfeeding represents an important route of repeated maternal microbial and ecological input. Culture-based, viability-based, and sequencing studies have demonstrated the presence of viable microorganisms and diverse microbial signals in the milk of healthy women, including lactic acid bacteria and bifidobacteria (Soto et al., 2014; Liu et al., 2020; El-Sayed et al., 2022). These microorganisms may originate from several interconnected maternal and infant niches, including the nipple and areolar skin, retrograde transfer from the infant oral cavity during breastfeeding, and a proposed maternal gut-to-mammary pathway. However, the existence and relative contribution of the entero-mammary route remain incompletely established (Rodríguez, 2014; Kordy et al., 2020; Ma et al., 2026).

Paired studies have demonstrated substantial taxonomic overlap between breast milk and infant fecal microbiota, while strain-resolved analyses have identified shared maternal–infant bacterial strains (Asnicar et al., 2017; Pannaraj et al., 2017; Feehily et al., 2023). Unlike the brief exposure associated with delivery, breastfeeding repeatedly introduces milk- and breast skin-associated microorganisms over the first weeks and months of life. Nevertheless, recent strain-resolved evidence suggests that breast milk is not a major reservoir of the dominant strains that persist in the infant gut, indicating that its effects may depend substantially on repeated exposure and ecological modulation rather than direct strain transmission alone.

The influence of breast milk extends well beyond microbial transfer. Human milk oligosaccharides (HMOs) are major ecological substrates that selectively shape the infant gut microbiota. Most HMOs resist digestion by infant enzymes and reach the distal intestine, where they can be utilized by infant-adapted gut microorganisms, particularly *Bifidobacterium* (Sela and Mills, 2010; Hu et al., 2023; Lordan et al., 2024). Considerable interspecies and interstrain variation exists in HMO utilization capacity, enabling strains with appropriate metabolic capabilities to occupy specific nutritional niches and gain a competitive advantage (Matsuki et al., 2016; Lawson et al., 2020). The timing and order of strain arrival can further generate priority effects, while HMO-mediated selection modifies the competitive environment in which these effects occur (Ojima et al., 2022; Laursen and Roager, 2023). Secretory IgA, lactoferrin, lysozyme, and other antimicrobial and immunomodulatory components of human milk further regulate microbial growth and host–microbe interactions (Gopalakrishna et al., 2019; Johnson-Hence et al., 2023; Ma et al., 2026).

Breastfeeding can therefore be viewed as a dynamic process that integrates continued maternal microbial input with ecological selection. Through the combined delivery of microorganisms, nutritional substrates, and immune factors, breastfeeding helps determine which microorganisms expand and persist in the infant intestine. Large longitudinal studies have shown that breastfeeding is a major determinant of gut microbiota development during the first year of life, whereas cessation of breastfeeding and transition to complementary feeding are associated with increasing microbial diversity and progression toward a more complex and mature microbiota (Bäckhed et al., 2015; Stewart et al., 2018).

### 2.4 Maternal skin and oral microbial exposure

Maternal skin represents an additional source of microbial exposure during the postnatal period. Skin-to-skin contact, holding, and breastfeeding repeatedly expose infants to microorganisms from maternal skin, particularly the breast and areolar region. Source-tracking analyses have shown that the infant gut microbiota is more similar to the areolar skin microbiota of the infant’s own mother than to that of unrelated mothers, with approximately 10% of the infant fecal bacterial community during the first month of life estimated to originate from maternal areolar skin (Pannaraj et al., 2017). Larger multi-site studies similarly suggest that maternal skin participates in a multisource transmission network affecting microbial establishment across several infant body sites (Bogaert et al., 2023). Increased postnatal skin-to-skin contact has also been shown to alter infant gut microbiota development, although the extent to which this reflects direct microbial transmission remains uncertain (Eckermann et al., 2024).

The maternal oral microbiota likewise represents an important source of microbial exposure in early life, particularly in relation to the establishment of the infant oral microbiota. Multi-site mother–infant studies have shown substantial community similarity between the infant oral microbiota and the maternal oral, milk, and areolar microbiota, with Streptococcus among the major shared bacterial groups (Drell et al., 2017). A subsequent high-resolution analysis of 448 mother–infant pairs further showed that infants shared significantly more oral bacterial amplicon sequence variants (ASVs) with their biological mothers than with unrelated mothers, providing stronger support for maternal-to-infant transmission of oral bacteria (Kageyama et al., 2022).

However, frequent exposure to maternal skin- or oral-associated microorganisms does not necessarily translate into long-term colonization of the infant gut. Strain-resolved metagenomic studies have demonstrated that microorganisms originating from different maternal body sites differ markedly in their persistence within the infant. Strains derived from maternal skin and the vagina tend to exhibit more transient colonization, whereas maternal gut-derived strains show greater persistence in the infant intestinal environment (Ferretti et al., 2018). The maternal skin and oral microbiota should therefore be viewed as important components of the multisource maternal microbial transmission network in early life, while their contribution to infant gut microbiota establishment should be distinguished from the more persistent transmission and colonization of maternal gut-derived strains.

### 2.5 Environmental and potential intrauterine microbial exposure

Although maternal-to-infant microbial transmission represents a major route of microbial acquisition in early life, the infant gut microbiota is not derived exclusively from maternal sources. Household members, healthcare environments, and other social contacts can provide additional microbial inputs (Brooks et al., 2014; Dubois et al., 2024). For example, cesarean-born infants exhibit reduced transmission of maternal *Bacteroides* strains together with increased colonization by hospital-associated opportunistic bacteria, including *Enterococcus*, *Enterobacter*, and *Klebsiella*, suggesting that disruption of maternal microbial transmission may increase the relative contribution of environmental sources (Shao et al., 2019). As infants grow and their social networks expand, horizontal microbial transmission may become increasingly important. A recent strain-resolved longitudinal study demonstrated extensive infant-to-infant transmission of gut bacterial strains after entry into nursery settings, with transmitted strains accumulating with increasing duration of shared contact (Ricci et al., 2026). Thus, the developing infant gut represents an open ecological system shaped by both vertical maternal transmission and postnatal horizontal acquisition.

Whether microbial colonization begins before birth, however, remains highly controversial. Earlier studies reported bacterial DNA or other microbial signals in the placenta, amniotic fluid, fetal tissues, and meconium, leading to the proposal of an intrauterine microbiota (Xie et al., 2025a). However, these sites contain extremely low microbial biomass and are therefore particularly susceptible to contamination during sampling and laboratory processing. Studies incorporating rigorous negative controls have failed to identify reproducible microbial communities in amniotic fluid from uncomplicated term pregnancies (Lim et al., 2018), while convincing evidence for a stable resident placental microbiome remains lacking. Similarly, direct sampling of fetal meconium before birth did not detect microbial signals distinguishable from negative controls (Kennedy et al., 2021). A subsequent multidisciplinary reassessment concluded that convincing evidence for a stable, viable, and replicating microbiome in healthy fetal tissues remains lacking (Kennedy et al., 2023). Detection of microbial nucleic acids or other microbial signals in low-biomass intrauterine samples should therefore not be equated with true prenatal colonization.

Importantly, the absence of convincing evidence for a resident fetal microbiome does not imply that maternal microbial influences begin only after birth. Animal studies have shown that the maternal microbiota can alter metabolite profiles in maternal circulation, the placenta, and fetal tissues, thereby influencing fetal development through microbiota-dependent metabolites (Vuong et al., 2020; Husso et al., 2023). Human fetal intestinal studies have likewise identified microbiota-associated metabolites in utero with evidence consistent with maternal origin (Wang et al., 2025). Microbiota-derived extracellular vesicles have also been proposed as potential mediators of maternal–fetal molecular communication, although their origins, transplacental trafficking, and biological significance remain to be clarified (Kaisanlahti et al., 2023). Current evidence therefore supports a distinction between prenatal microbial influence and prenatal microbial colonization: maternal microbiota-derived metabolites and other molecular signals may affect the fetus before birth, whereas extensive acquisition and stable colonization by viable microorganisms appear to occur predominantly during and after delivery.

### 2.6 Ecological assembly and succession of the neonatal gut microbiota

The neonatal gut microbiota is not established through the simple accumulation of microorganisms encountered after birth, but through a dynamic ecological assembly process governed by dispersal, selection, drift, microbial interactions, and priority effects (Sprockett et al., 2018). Thus, microbial exposure does not necessarily lead to persistent colonization. Microorganisms must first reach the infant gut and subsequently overcome ecological filters imposed by the intestinal environment and resident community. Their colonization success therefore depends on both transmission and ecological fitness. Early-arriving microorganisms can further influence the establishment of later colonizers through priority effects, making early community assembly partly dependent on colonization history (Ojima et al., 2022).

Following birth, the infant gut microbiota undergoes rapid ecological succession. Early communities are relatively enriched in oxygen-tolerant and fast-growing microorganisms, followed by increasing dominance of anaerobic taxa as the intestinal environment becomes progressively more oxygen depleted (Guittar et al., 2019). Nutritional changes further shape this trajectory. During breastfeeding, HMOs selectively favor *Bifidobacterium* species and strains capable of utilizing these substrates, while differences in strain arrival time and metabolic capacity can modify competitive and priority effects (Lawson et al., 2020; Ojima et al., 2022; Laursen and Roager, 2023). With decreasing breastfeeding and increasing dietary complexity, the gut microbiota gradually progresses toward a more diverse and mature community structure (Bäckhed et al., 2015; Stewart et al., 2018).

Because microbial assembly is strongly time dependent, disturbances during critical early-life windows can redirect subsequent developmental trajectories. Cesarean delivery may reduce the acquisition of maternally derived commensals, whereas antibiotic exposure can decrease microbial and strain-level diversity, destabilize community structure, and delay microbiota maturation (Bokulich et al., 2016; Yassour et al., 2016; Shao et al., 2019). Early-life dysbiosis should therefore be viewed not simply as altered abundance of individual taxa, but as a disruption of microbial transmission, strain establishment, and ecological succession.

Importantly, delivery-associated microbial differences should be interpreted in an age-dependent context. Some differences may be most pronounced during early infancy and diminish as the gut microbiota matures and dietary exposures change, whereas others may persist beyond infancy. These patterns should be distinguished using longitudinal studies, and persistence alone should not be taken as evidence of microbial dysfunction or adverse health outcomes. Determining whether transient or persistent differences are associated with altered microbial functions or clinically meaningful outcomes is essential for establishing which microbial features, if any, warrant intervention.

## 3 Disruption of maternal microbial transmission

Maternal-to-infant microbial transmission during the perinatal period can be disrupted by several common obstetric and neonatal exposures, particularly cesarean delivery, preterm birth, and antibiotic use. Cesarean-delivered infants exhibit altered gut microbiota development characterized by reduced vertical transmission of maternal gut-associated microorganisms, particularly *Bacteroides*, delayed acquisition of key anaerobic commensals, and increased colonization by opportunistic and hospital-associated taxa such as *Enterococcus*, *Enterobacter*, and *Klebsiella* (Jakobsson et al., 2014; Rutayisire et al., 2016; Shao et al., 2019; Coker et al., 2021). These findings indicate that cesarean delivery disrupts not only early microbial composition but also the transfer and establishment of maternally derived, gut-adapted strains that contribute to normal intestinal community assembly.

Preterm birth is likewise associated with profound disruption of early gut microbiota development. Earlier gestational age is associated with delayed microbiota maturation, reduced colonization by *Bifidobacterium* and *Bacteroides*, and increased abundance of opportunistic taxa, including *Enterobacter*, *Enterococcus*, *Corynebacterium*, *Klebsiella*, and *Clostridium* (Mai et al., 2013; Drell et al., 2014; Arboleya et al., 2015; Xie et al., 2025b). These alterations likely reflect both developmental immaturity of the intestinal ecosystem and greater exposure to neonatal intensive care environments, where antibiotic treatment, delayed enteral feeding, and reduced contact with maternal microbial reservoirs may further limit the acquisition and persistence of maternally transmitted strains. Preterm infants also exhibit altered production of microbiota-derived metabolites, including short-chain fatty acids, which may further affect intestinal barrier and immune maturation (Bergström et al., 2014; Aguilar-Lopez et al., 2021).

Antibiotic exposure represents another major ecological disturbance during this critical period of microbial establishment. Prenatal, intrapartum, and early postnatal antibiotics may interfere with maternal microbial transmission at multiple stages by perturbing the maternal microbial reservoir, reducing the availability of transmissible commensals, and impairing the establishment of newly acquired strains in the infant gut. Antibiotic exposure can suppress susceptible commensals while favoring antibiotic-tolerant and opportunistic microorganisms (Zou et al., 2018). Intrapartum antibiotic exposure has been associated with depletion of *Actinobacteria*, including *Bifidobacteriaceae*, and expansion of *Proteobacteria*, whereas early postnatal antibiotic treatment has been linked to reduced *Lactobacillus* and *Bifidobacterium* and increased *Enterobacteriaceae* (Arboleya et al., 2015; Zhou et al., 2020; Zimmermann and Curtis, 2020). Such perturbations may therefore alter not only community composition but also the ecological conditions required for the successful engraftment of maternally derived strains.

Importantly, these exposures frequently overlap and may act synergistically rather than independently. Preterm infants, for example, are more likely to be delivered by cesarean section, receive broad-spectrum antibiotics, require neonatal intensive care, experience delayed enteral feeding, and have reduced contact with maternal microbial reservoirs. Their combined effects may redirect early gut microbiota assembly away from maternally derived, gut-adapted strains toward opportunistic and healthcare-associated microorganisms, thereby delaying ecological and functional maturation. From an ecological perspective, cesarean delivery, preterm birth, and antibiotic exposure can therefore be viewed not simply as determinants of altered taxonomic composition, but as disruptions of microbial transmission, ecological selection, and subsequent strain engraftment. Restoring these disrupted processes provides the biological rationale for interventions aimed at re-establishing normal early-life microbiota development.

## 4 Current strategies for restoring early-life microbiota

Disruption of maternal microbial transmission and early gut microbiota assembly has prompted increasing interest in strategies aimed at modifying microbiota development in infants exposed to cesarean delivery, antibiotics, or other perinatal perturbations. Current approaches range from supplementation with selected beneficial microorganisms to the transfer of maternal microbial communities. Although these interventions can modify early gut microbiota composition, they differ in their mechanisms of action, microbial sources, safety profiles, and levels of evidence for clinical benefit. Importantly, greater microbial diversity, more extensive microbial transfer, or more persistent engraftment should not be assumed to confer greater health benefits. Microbial diversity and community composition should instead be interpreted in the context of infant age, feeding practices, microbial function, and relevant clinical outcomes. Accordingly, these interventions should be evaluated not simply by their ability to alter microbiota composition or promote similarity to a reference population, but by their safety and their demonstrated effects on microbial function and clinically meaningful infant health outcomes (Figure 2).

**Figure 2:**
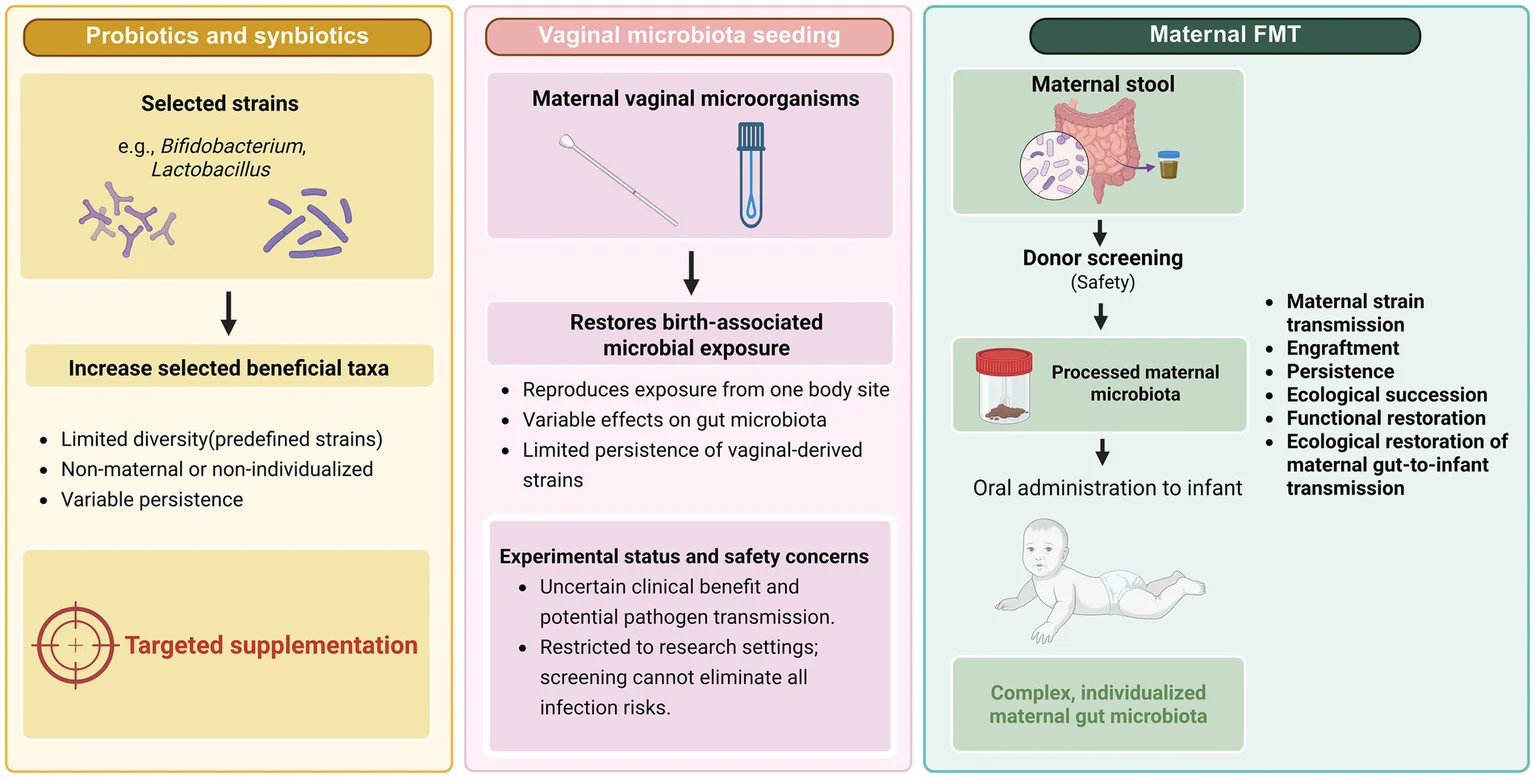
Conceptual comparison of current strategies for restoring the early-life microbiota. Probiotics and synbiotics provide selected microbial strains as targeted supplementation, whereas vaginal microbiota seeding aims to reproduce microbial exposure associated with vaginal delivery. Maternal FMT represents a distinct ecological restoration strategy that transfers a complex, individualized maternal gut microbial community, potentially supporting maternal-to-infant microbial transfer. Potential effects under investigation include donor-strain engraftment and persistence, modulation of microbial succession and function, and enhanced colonization resistance. Vaginal microbiota seeding and maternal FMT remain investigational, and their long-term safety and clinical benefits require further evaluation.

### 4.1 Probiotics and synbiotics

Probiotics and synbiotics represent widely studied approaches for modifying the infant gut microbiota, most commonly using *Bifidobacterium* and lactic acid bacteria with or without prebiotic substrates. Clinical studies have shown that these interventions can increase bifidobacterial abundance and partially modify microbiota composition in cesarean-born, antibiotic-exposed, and preterm infants (Bazanella et al., 2017; Chua et al., 2017; Korpela et al., 2018; van Best et al., 2020; Zhong et al., 2021). For example, supplementation with *Bifidobacterium breve* M-16 V and galacto−/fructo-oligosaccharides increased bifidobacteria and reduced Enterobacteriaceae in formula-fed cesarean-born infants (Chua et al., 2017), while multispecies probiotics attenuated microbiota alterations associated with cesarean delivery or antibiotic exposure (Korpela et al., 2018).

However, successful administration does not necessarily result in persistent engraftment, and colonization depends strongly on the administered strain and the ecological context of the recipient gut. Some bifidobacterial strains show limited persistence after supplementation, whereas infant-adapted strains such as *Bifidobacterium longum* subsp. infantis can establish more sustained colonization in breastfed infants, likely reflecting their capacity to utilize human milk oligosaccharides (Frese et al., 2017; O’Brien et al., 2022). These observations highlight that probiotic engraftment is strain- and niche-dependent rather than universally sustained.

More importantly, conventional probiotic and synbiotic formulations contain only a limited number of predefined strains and therefore cannot reproduce the diversity and maternal specificity of naturally transmitted gut microbial communities. Given that the maternal gut represents a major reservoir of persistent infant gut strains (Sinha et al., 2026), probiotics and synbiotics are better regarded as targeted microbial supplementation rather than restoration of maternal microbial transmission. Although they can promote selected beneficial microorganisms and functions, their capacity to restore the broader strain-level assembly and succession of the infant gut remains limited.

### 4.2 Vaginal microbiota seeding

Vaginal microbiota seeding was developed from the hypothesis that bypassing the birth canal during cesarean delivery deprives newborns of microbial exposures normally encountered during vaginal birth. The approach typically involves exposing cesarean-born infants to maternal vaginal fluids shortly after delivery. An early pilot study showed that vaginal microbial transfer partially restored vaginal-associated microorganisms across infant skin, oral, and gut communities during the first month of life, providing initial evidence that birth-associated microbial exposure could be experimentally modified (Dominguez-Bello et al., 2016).

Subsequent randomized studies, however, have produced inconsistent results. Oral administration of maternal vaginal microorganisms did not significantly alter early gut microbiome development (Wilson et al., 2021), whereas vaginal seeding increased maternal microbial engraftment and altered neonatal skin and stool microbiota in another randomized trial (Mueller et al., 2023). A separate trial found no significant effects on infant gut microbiota, growth, or allergy risk during the first 2 years of life (Liu et al., 2023), while a blinded randomized trial reported accelerated gut microbiota maturation and changes in fecal metabolites and metabolic functions following vaginal microbiota transfer (Zhou et al., 2023). Collectively, these findings suggest that vaginal microbial transfer can modify early microbial exposure, but its effects on gut microbiota development and longer-term clinical outcomes remain variable.

A more fundamental limitation is that exposure to vaginal microorganisms does not necessarily reproduce the microorganisms most likely to persist in the infant intestine. Recent strain-resolved evidence indicates that the maternal gut is the predominant reservoir of infant gut strains, whereas transmission from the maternal vagina occurs only occasionally (Sinha et al., 2026). Vaginal seeding should therefore be viewed primarily as an attempt to restore delivery-associated microbial exposure, rather than as a complete reconstruction of maternal gut-to-infant microbial transmission.

### 4.3 Limitations and the need for maternal gut microbiota restoration

The limitations of probiotics, synbiotics, and vaginal seeding highlight an important distinction between microbial supplementation and ecological restoration. Probiotics introduce a limited number of selected strains, whereas vaginal seeding attempts to reproduce one component of microbial exposure during birth. Neither approach fully recreates the diverse and individualized repertoire of maternal gut-derived strains that contributes substantially to persistent infant intestinal colonization. Large-scale strain-resolved analyses indicate that maternal gut strain transmission is time dependent, is more likely when the corresponding species is abundant in the maternal gut, and is associated with greater persistence of maternally shared strains during infancy (Sinha et al., 2026).

Vaginal seeding also faces important safety and translational limitations. Maternal vaginal secretions may contain potentially pathogenic microorganisms that can be transmitted to the neonate. The American College of Obstetricians and Gynecologists (ACOG) therefore does not recommend vaginal seeding outside institutional review board-approved research protocols until adequate evidence regarding its safety and benefits becomes available. The American Academy of Pediatrics (AAP) similarly advises against its routine use outside research settings because of the potential transmission of pathogens, including group B *Streptococcus* and herpes simplex virus (Nolt et al., 2022; Vaginal Seeding | ACOG, 2017). These safety concerns, together with inconsistent microbiome and clinical outcomes, limit the current applicability of vaginal seeding as a strategy for restoring disrupted maternal microbial transmission.

Source-resolved studies further support the maternal gut as a major reservoir of persistent infant colonizers. Maternal vaginal microbiota shows relatively limited overlap with infant fecal communities compared with maternal rectal microbiota (Sakwinska et al., 2017). In a longitudinal multi-site metagenomic study, the maternal gut accounted for an estimated 22.1% of microbial abundance in the infant gut, compared with 16.3% from the vagina, 7.2% from the oral cavity, and 5.0% from the skin; importantly, maternal gut-derived strains showed greater persistence, whereas vaginal- and skin-derived strains were generally more transient (Ferretti et al., 2018). More recent strain-resolved evidence similarly identifies the maternal gut as the predominant reservoir of persistent infant gut strains.

Maternal FMT represents a distinct approach because it transfers a complex maternal gut microbial community rather than a limited number of selected microorganisms or microorganisms from a single maternal body site. This approach may facilitate the transfer of multiple maternal gut-derived strains and their associated ecological interactions. However, the greater complexity of the transferred community does not necessarily translate into improved microbial function or infant health. Compared with more targeted interventions, maternal FMT also presents distinct safety challenges, including the potential transmission of pathogens or undesirable microbial traits and uncertainty regarding the long-term effects of transferred microorganisms on the developing infant microbiota. Therefore, the potential value of maternal FMT should be evaluated in terms of its safety, the functional consequences of microbial engraftment, and clinically meaningful outcomes, rather than the diversity or complexity of the transferred community alone.

## 5 FMT as an ecological restoration strategy

### 5.1 Rationale for FMT

The rationale for maternal FMT is supported by evidence of natural mother-to-infant microbial transmission. Strain-resolved metagenomic studies have demonstrated maternal strain sharing in early-life gut colonization, highlighting the selective nature of microbial transmission (Asnicar et al., 2017; Ferretti et al., 2018; Sprockett et al., 2018; Yassour et al., 2018). Culture- and metagenomics-based analyses have further documented strain sharing of Bifidobacterium and Bacteroides between mothers and infants (Feehily et al., 2023).

Maternal gut-derived strains can persist in the infant intestine, although transmission and persistence vary across microbial sources and strains (Ferretti et al., 2018; Lou et al., 2021). Population-scale analyses have documented mother–infant gut strain sharing during the first year of life (Valles-Colomer et al., 2023). A longitudinal study of 714 mother–infant pairs further identified the maternal gut as a major reservoir of infant gut strains, with transmission associated with maternal species abundance and greater persistence of shared strains (Sinha et al., 2026).

However, microbial exposure does not guarantee successful transmission or persistent colonization. In the MicrobeMom randomized trial, the supplemented *Bifidobacterium breve* 702258 strain was detected in only 2 of 65 infants born to mothers receiving the probiotic, illustrating the challenges of achieving maternal-to-infant transmission of an administered strain (Moore et al., 2023). These findings suggest that microbial establishment depends on multiple factors, including strain characteristics, host conditions, and the intestinal ecological environment.

Cesarean delivery can alter early microbial acquisition, including the transmission of maternal gut-associated commensals, motivating strategies to restore maternal microbial transfer (Hourigan et al., 2022; Liu et al., 2024). Maternal FMT is one such investigational approach, transferring a complex microbial community from the infant’s own mother rather than a limited number of selected strains or microorganisms from a single maternal body site. Whether this approach can improve persistent colonization, restore relevant microbial functions, and translate into clinically meaningful benefits remains to be established. Initial intervention studies provide proof-of-concept support for further investigation, but rigorous evaluation of safety and clinical efficacy is needed before broader application.

### 5.2 Current evidence

The first direct human evidence for maternal FMT in newborns was provided by Korpela et al. in a proof-of-concept study of term infants born by cesarean section. Of 17 mothers screened, only 7 fulfilled the stringent donor eligibility criteria. Their infants received a small amount of carefully screened maternal fecal microbiota orally shortly after birth. Maternal FMT rapidly altered gut microbiota development, and from approximately the first week of life, the microbiota of treated cesarean-born infants developed toward a pattern more similar to that observed in vaginally delivered infants than in untreated cesarean-born controls. The intervention also restored characteristic cesarean-associated deficiencies in taxa such as Bacteroides and reduced the representation of potential pathobionts commonly enriched after cesarean birth (Korpela et al., 2020).

Subsequent strain-resolved metagenomic analysis strengthened these findings by showing that the observed changes reflected increased transmission of maternal bacterial strains rather than merely taxonomic convergence. Dubois et al. (2024) demonstrated that maternal FMT increased mother–infant strain sharing in cesarean-born infants, increased the empirical growth rates of transmitted bacteria, and reduced colonization by potential pathogens. These findings provide direct strain-level evidence that maternal FMT can restore aspects of the maternal microbial seeding process disrupted by cesarean delivery.

The evidence base nevertheless remains limited. The SECFLOR study was designed as a double-blind, randomized, placebo-controlled trial to determine whether maternal FMT can restore microbiota development and influence immune maturation in term infants born by elective cesarean section, with longitudinal microbiome, immunological, growth, and clinical assessments extending to 24 months (Carpén et al., 2022). A subsequent conference report described 31 randomized infants; three adverse events occurred in the FMT group, but none was judged to be related to the intervention. Complete peer-reviewed microbiome and immunological efficacy results were not provided in that report (Carpén et al., 2025).

Reviews of maternal microbial restoration strategies similarly emphasize that most available intervention studies remain relatively small and that evidence for durable clinical benefits is still limited (Jurgiel et al., 2026). Thus, current human evidence supports the microbiological feasibility and ecological plausibility of maternal FMT, but remains insufficient to establish long-term clinical efficacy.

### 5.3 Microbial engraftment and potential benefits

A central question is whether maternally transferred microorganisms merely pass transiently through the infant gut or establish persistent populations. In natural mother-to-infant transmission, strain identity rather than genus- or species-level similarity provides the strongest evidence of direct microbial transmission (Yassour et al., 2018). Maternal origin is also associated with persistence: maternal gut-derived strains are significantly more likely than non-maternal strains to remain detectable during infancy, and traits related to carbohydrate utilization, surface adhesion, and iron acquisition have been associated with persistence (Lou et al., 2021). Large-scale longitudinal data further indicate that maternally shared strains are more likely to persist to later infancy (Sinha et al., 2026).

For maternal FMT, successful engraftment should therefore be distinguished from short-term donor-strain detection. Ideally, engraftment should refer to donor-derived strains that establish in the recipient ecosystem and remain detectable over time. This principle is consistent with strain-resolved studies of conventional FMT in adults, which have demonstrated that donor and recipient strains can coexist after transplantation, although the applicability of these findings to maternal FMT in newborns remains uncertain (Li et al., 2016). In cesarean-born infants, Dubois et al. provided direct strain-level evidence that maternal FMT increases mother–infant strain sharing and promotes growth of transmitted bacterial populations (Dubois et al., 2024).

Engraftment is unlikely to depend on exposure alone. Natural transmission studies indicate that maternal abundance, strain-specific ecological traits, delivery mode, breastfeeding, and compatibility with the infant intestinal environment all influence transmission and persistence (Li et al., 2016). PubMed In the context of maternal FMT, timing and dose of administration, antibiotic exposure, breastfeeding, donor microbiota composition, and competition with previously established microorganisms are also likely to influence engraftment, although these determinants remain to be systematically defined.

The potential ecological benefits of successful engraftment extend beyond increasing the abundance of individual taxa. Re-establishment of maternal gut-adapted strains may facilitate recovery of early-life commensals, restore microbial interactions and metabolic functions, and strengthen colonization resistance against opportunistic microorganisms. Consistent with this possibility, maternal FMT restored cesarean-associated microbiota alterations in the initial proof-of-concept study, while subsequent strain-resolved analysis demonstrated increased maternal strain sharing and reduced pathogen colonization (Korpela et al., 2020; Dubois et al., 2024).

However, these microbiological effects should not yet be interpreted as established clinical benefits. Evidence that maternal FMT improves immune maturation, reduces infection or allergic disease, or influences longer-term metabolic or neurodevelopmental outcomes remains insufficient (Jurgiel et al., 2026). Changes in microbiota composition, increased maternal–infant strain sharing, or greater similarity to the microbiota of vaginally delivered infants do not, by themselves, establish improved infant health. Similarly, greater microbial diversity or more extensive strain engraftment should not be considered surrogate markers of clinical benefit unless their predictive value has been validated. Evaluation of maternal FMT should therefore prioritize safety, including adverse events and the potential for pathogen transmission, alongside prespecified clinical outcomes relevant to infant health. Longitudinal follow-up is also needed to determine whether transferred strains persist, whether microbiological changes have beneficial or adverse functional consequences, and whether these changes are associated with meaningful health outcomes. Accordingly, maternal FMT should remain an investigational strategy until its safety and clinical benefits are established. Its success should be assessed through an integrated evaluation of safety, strain-level engraftment, microbial function, developmental trajectories, and clinically meaningful outcomes, rather than by taxonomic similarity to vaginally delivered infants or microbial diversity alone. Current evidence is insufficient to establish its clinical superiority over probiotics, synbiotics, or other microbiota-targeted interventions.

## 6 Safety and translational challenges

Despite its ecological rationale, maternal FMT presents substantial safety and translational challenges because the recipients are newborns, many of whom are otherwise healthy. Biological relatedness does not guarantee donor safety. In the initial proof-of-concept study, only 7 of 17 screened mothers met eligibility criteria, highlighting the need for rigorous clinical and microbiological screening before maternal stool is administered to an infant (Korpela et al., 2020). Experience with adult FMT has demonstrated transmission of antimicrobial-resistant and pathogenic bacteria, including extended-spectrum β-lactamase-producing *Escherichia coli* and Shiga toxin-producing *E. coli* (DeFilipp et al., 2019; Zellmer et al., 2021). Accordingly, neonatal applications require stringent screening for enteric pathogens and antimicrobial-resistant organisms. A comprehensive screening strategy should also assess relevant medical and infectious histories and, where appropriate, screen for blood-borne infections, intestinal parasites, and other transmissible pathogens. Screening criteria should be predefined and updated as new safety risks emerge, and donor eligibility should be confirmed before each donation rather than assumed from previous screening results.

Clinical translation also requires standardization of stool collection, processing, dose, storage, microbial viability, timing, and route of administration. Published neonatal protocols have used small quantities of carefully screened maternal fecal material administered orally with human milk shortly after cesarean delivery, but the optimal dose, formulation, and dosing schedule remain uncertain (Korpela et al., 2020; Helve et al., 2021; Carpén et al., 2022). Key procedural parameters that should be explicitly reported include the interval between stool collection and processing, the diluent and any cryoprotectant used, homogenization and filtration procedures, storage temperature and duration, and the conditions used for thawing and administration. The administered dose should be reported using a clearly defined measure, together with the preparation volume and the infant’s age at administration. In addition, studies should specify whether the material is administered with human milk, the precise oral administration procedure, and the interval between birth and dosing. Standardized manufacturing procedures, product traceability, predefined release criteria, and consistent definitions of microbial engraftment will therefore be essential for comparing outcomes across trials. Where available, published protocols should also be accompanied by information on product quality control, including microbiological testing before administration and documentation of batch identity and handling conditions.

Recipient selection is equally important. Existing studies have largely focused on healthy term infants born by elective cesarean section, and their findings cannot be directly extrapolated to preterm infants, infants requiring intensive care, or those with underlying disease. Although these populations may experience more profound disruption of maternal microbial transmission, they may also be more vulnerable to invasive infection or unintended donor-strain colonization. Maternal FMT should therefore be considered a targeted rather than universal intervention. Post-administration monitoring should include systematic recording of immediate reactions and adverse events, feeding tolerance, suspected or confirmed infections, growth, and relevant clinical outcomes. Longer-term assessments may additionally examine microbiome development, immune maturation, and the occurrence of allergic, metabolic, or other health conditions, while distinguishing prespecified study outcomes from exploratory endpoints.

Long-term safety remains unresolved. Although no apparent clinical adverse events were reported during the short follow-up of the initial proof-of-concept study, current evidence is insufficient to determine longer-term health benefits or rare delayed risks (Jurgiel et al., 2026). Because donor-derived strains may persist after transplantation, extended follow-up of microbiome development, immune function, growth, and clinical outcomes is warranted; randomized trial protocols have incorporated such assessments through early childhood (Carpén et al., 2022). Follow-up protocols should define the timing of clinical assessments, procedures for investigating suspected infections or other adverse events, and criteria for evaluating the persistence of potentially undesirable donor-derived organisms. Importantly, protocol-defined monitoring plans should not be interpreted as evidence that long-term safety or clinical benefit has already been demonstrated. Given that the intervention modifies a developing microbial ecosystem in newborns who cannot provide consent, maternal FMT should remain confined to rigorously controlled clinical research until its safety and long-term benefits are established.

Importantly, the current evidence is largely derived from carefully selected healthy term infants born by elective cesarean section, and its applicability to other neonatal populations remains uncertain. In particular, the safety, feasibility, and potential benefits of maternal FMT cannot be extrapolated to preterm infants, infants receiving antibiotic treatment, or those requiring intensive care, as these populations may differ substantially in microbial community development, immune status, clinical vulnerability, and exposure to medical interventions. These groups should therefore be evaluated separately in appropriately designed clinical studies before maternal FMT is considered for clinical use.

## 7 Future perspectives and conclusions

Maternal FMT represents a distinct approach to early-life microbiota restoration because it seeks to re-establish disrupted maternal gut-to-infant microbial transmission rather than simply supplement selected beneficial taxa. Initial proof-of-concept evidence indicates that carefully screened maternal fecal microbiota can redirect gut microbiota development in cesarean-born infants toward patterns more similar to those observed after vaginal delivery (Korpela et al., 2020). However, current evidence remains limited by small study populations, heterogeneous intervention approaches, short follow-up, and a predominance of microbiome rather than clinical outcomes.

Future studies should therefore move beyond broad taxonomic similarity and determine which maternal strains successfully engraft, how long they persist, and whether they restore microbial functions and developmental trajectories characteristic of normal early life. Strain-resolved metagenomics integrated with metabolomics, immune profiling, and longitudinal clinical phenotyping will be particularly important. Successful restoration should ultimately be defined not simply by increases in *Bifidobacterium* or *Bacteroides*, but by recovery of appropriate microbial succession, ecological function, and host–microbiota interactions.

Whole-stool maternal FMT may ultimately serve as a proof-of-principle rather than the final therapeutic product. As the determinants of maternal strain transmission and persistence become better defined, future approaches may shift toward cultured maternal strain consortia, defined microbial communities, or other personalized microbial products that preserve the ecological advantages of maternal matching while improving safety and standardization. Maternal FMT should therefore currently be regarded as an investigational strategy, but it may provide an important conceptual foundation for the development of safe and precise early-life microbiota engineering.

## References

[ref1] ACOG (2017) Vaginal Seeding. Available online at: https://www.acog.org/clinical/clinical-guidance/committee-opinion/articles/2017/11/vaginal-seeding?utm_source=chatgpt.com (Accessed May 16, 2026).

[ref2] Aguilar-Lopez M. Dinsmoor A. M. Ho T. T. B. Donovan S. M. (2021). A systematic review of the factors influencing microbial colonization of the preterm infant gut. Gut Microbes 13, 1–33. doi: 10.1080/19490976.2021.1884514, 33818293 PMC8023245

[ref3] Arboleya S. Sánchez B. Milani C. Duranti S. Solís G. Fernández N. . (2015). Intestinal microbiota development in preterm neonates and effect of perinatal antibiotics. J. Pediatr. 166, 538–544. doi: 10.1016/j.jpeds.2014.09.041, 25444008

[ref4] Asnicar F. Manara S. Zolfo M. Truong D. T. Scholz M. Armanini F. . (2017). Studying vertical microbiome transmission from mothers to infants by strain-level metagenomic profiling. mSystems 2:e00164-16. doi: 10.1128/mSystems.00164-16, 28144631 PMC5264247

[ref5] Bäckhed F. Roswall J. Peng Y. Feng Q. Jia H. Kovatcheva-Datchary P. . (2015). Dynamics and stabilization of the human gut microbiome during the first year of life. Cell Host Microbe 17, 690–703. doi: 10.1016/j.chom.2015.04.004, 25974306

[ref6] Bazanella M. Maier T. V. Clavel T. Lagkouvardos I. Lucio M. Maldonado-Gòmez M. X. . (2017). Randomized controlled trial on the impact of early-life intervention with bifidobacteria on the healthy infant fecal microbiota and metabolome. Am. J. Clin. Nutr. 106, 1274–1286. doi: 10.3945/ajcn.117.157529, 28877893

[ref7] Bergström A. Skov T. H. Bahl M. I. Roager H. M. Christensen L. B. Ejlerskov K. T. . (2014). Establishment of intestinal microbiota during early life: a longitudinal, explorative study of a large cohort of Danish infants. Appl. Environ. Microbiol. 80, 2889–2900. doi: 10.1128/AEM.00342-14, 24584251 PMC3993305

[ref8] Bogaert D. van Beveren G. J. de Koff E. M. Lusarreta Parga P. Balcazar Lopez C. E. Koppensteiner L. . (2023). Mother-to-infant microbiota transmission and infant microbiota development across multiple body sites. Cell Host Microbe 31, 447–460.e6. doi: 10.1016/j.chom.2023.01.018, 36893737

[ref9] Bokulich N. A. Chung J. Battaglia T. Henderson N. Jay M. Li H. . (2016). Antibiotics, birth mode, and diet shape microbiome maturation during early life. Sci. Transl. Med. 8:343ra82. doi: 10.1126/scitranslmed.aad7121, 27306664 PMC5308924

[ref10] Brooks B. Firek B. A. Miller C. S. Sharon I. Thomas B. C. Baker R. . (2014). Microbes in the neonatal intensive care unit resemble those found in the gut of premature infants. Microbiome 2:1. doi: 10.1186/2049-2618-2-1, 24468033 PMC4392516

[ref11] Carpén N. Brodin P. de Vos W. M. Salonen A. Kolho K.-L. Andersson S. . (2022). Transplantation of maternal intestinal flora to the newborn after elective cesarean section (SECFLOR): study protocol for a double blinded randomized controlled trial. BMC Pediatr. 22:565. doi: 10.1186/s12887-022-03609-3, 36175995 PMC9521560

[ref12] Carpén N. K. de Vos W. M. Salonen A. Kolho K.-L. Andersson S. Helve O. (2025). 381. Transplantation of maternal intestinal flora to the newborn after elective cesarean section - a randomized controlled trial (SECFLOR). Open Forum Infect. Dis. 12:ofae631.012. doi: 10.1093/ofid/ofae631.012 PMC9521560 36175995

[ref13] Chua M. C. Ben-Amor K. Lay C. Neo A. G. E. Chiang W. C. Rao R. . (2017). Effect of synbiotic on the gut microbiota of cesarean delivered infants: a randomized, double-blind, multicenter study. J. Pediatr. Gastroenterol. Nutr. 65, 102–106. doi: 10.1097/MPG.0000000000001623, 28644357

[ref14] Coker M. O. Laue H. E. Hoen A. G. Hilliard M. Dade E. Li Z. . (2021). Infant feeding alters the longitudinal impact of birth mode on the development of the gut microbiota in the first year of life. Front. Microbiol. 12:642197. doi: 10.3389/fmicb.2021.642197, 33897650 PMC8059768

[ref15] DeFilipp Z. Bloom P. P. Torres Soto M. Mansour M. K. Sater M. R. A. Huntley M. H. . (2019). Drug-resistant *E. coli* bacteremia transmitted by fecal microbiota transplant. N. Engl. J. Med. 381, 2043–2050. doi: 10.1056/NEJMoa1910437 31665575

[ref16] Dominguez-Bello M. G. Costello E. K. Contreras M. Magris M. Hidalgo G. Fierer N. . (2010). Delivery mode shapes the acquisition and structure of the initial microbiota across multiple body habitats in newborns. Proc. Natl. Acad. Sci. 107, 11971–11975. doi: 10.1073/pnas.1002601107, 20566857 PMC2900693

[ref17] Dominguez-Bello M. G. Jesus-Laboy K. M. D. Shen N. Cox L. M. Amir A. Gonzalez A. . (2016). Partial restoration of the microbiota of cesarean-born infants via vaginal microbial transfer. Nat. Med. 22:250. doi: 10.1038/nm.4039 PMC5062956 26828196

[ref18] Dos Santos S. J. Shukla I. Hill J. E. Money D. M. and Maternal Microbiome Legacy Project Team (2023). Birth mode does not determine the presence of shared bacterial strains between the maternal vaginal microbiome and the infant stool microbiome. Microbiol. Spectrum 11:e0061423. doi: 10.1128/spectrum.00614-23 PMC10433807 37338388

[ref19] Drell T. Lutsar I. Stšepetova J. Parm U. Metsvaht T. Ilmoja M.-L. . (2014). The development of gut microbiota in critically ill extremely low birth weight infants assessed with 16S rRNA gene based sequencing. Gut Microbes 5, 304–503. doi: 10.4161/gmic.28849, 25184833 PMC4153767

[ref20] Drell T. Štšepetova J. Simm J. Rull K. Aleksejeva A. Antson A. . (2017). The influence of different maternal microbial communities on the development of infant gut and Oral microbiota. Sci. Rep. 7:9940. doi: 10.1038/s41598-017-09278-y, 28855595 PMC5577157

[ref21] Dubois L. Valles-Colomer M. Ponsero A. Helve O. Andersson S. Kolho K.-L. . (2024). Paternal and induced gut microbiota seeding complement mother-to-infant transmission. Cell Host Microbe 32, 1011–1024.e4. doi: 10.1016/j.chom.2024.05.004, 38870892

[ref22] Eckermann H. A. Meijer J. Cooijmans K. Lahti L. de Weerth C. (2024). Daily skin-to-skin contact alters microbiota development in healthy full-term infants. Gut Microbes 16:2295403. doi: 10.1080/19490976.2023.2295403, 38197254 PMC10793693

[ref23] El-Sayed A. I. M. El-Borai A. M. Akl S. H. El-Aassar S. A. Abdel-Latif M. S. (2022). Identification of lactobacillus strains from human mother milk and cottage cheese revealed potential probiotic properties with enzymatic activity. Sci. Rep. 12:22522. doi: 10.1038/s41598-022-27003-2, 36581674 PMC9800376

[ref24] Feehily C. O’Neill I. J. Walsh C. J. Moore R. L. Killeen S. L. Geraghty A. A. . (2023). Detailed mapping of Bifidobacterium strain transmission from mother to infant via a dual culture-based and metagenomic approach. Nat. Commun. 14:3015. doi: 10.1038/s41467-023-38694-0, 37230981 PMC10213049

[ref25] Ferretti P. Pasolli E. Tett A. Asnicar F. Gorfer V. Fedi S. . (2018). Mother-to-infant microbial transmission from different body sites shapes the developing infant gut microbiome. Cell Host Microbe 24, 133–145.e5. doi: 10.1016/j.chom.2018.06.005, 30001516 PMC6716579

[ref26] Frese S. A. Hutton A. A. Contreras L. N. Shaw C. A. Palumbo M. C. Casaburi G. . (2017). Persistence of supplemented *Bifidobacterium longum* subsp. *infantis* EVC001 in breastfed infants. mSphere 2:e00501-17. doi: 10.1128/mSphere.00501-17, 29242832 PMC5717325

[ref27] Gopalakrishna K. P. Macadangdang B. R. Rogers M. B. Tometich J. T. Firek B. A. Baker R. . (2019). Maternal IgA protects against the development of necrotizing enterocolitis in preterm infants. Nat. Med. 25, 1110–1115. doi: 10.1038/s41591-019-0480-9, 31209335 PMC7424541

[ref28] Gregory K. E. Samuel B. S. Houghteling P. Shan G. Ausubel F. M. Sadreyev R. I. . (2016). Influence of maternal breast milk ingestion on acquisition of the intestinal microbiome in preterm infants. Microbiome 4:68. doi: 10.1186/s40168-016-0214-x, 28034306 PMC5200970

[ref29] Guittar J. Shade A. Litchman E. (2019). Trait-based community assembly and succession of the infant gut microbiome. Nat. Commun. 10:512. doi: 10.1038/s41467-019-08377-w, 30710083 PMC6358638

[ref30] He P. Yu L. Tian F. Chen W. Zhang H. Zhai Q. (2024). Effects of probiotics on preterm infant gut microbiota across populations: a systematic review and meta-analysis. Advances in nutrition (Bethesda, Md.) 15:100233. doi: 10.1016/j.advnut.2024.100233, 38908894 PMC11251410

[ref31] Helve O. Dikareva E. Stefanovic V. Kolho K.-L. Salonen A. de Vos W. M. . (2021). Protocol for oral transplantation of maternal fecal microbiota to newborn infants born by cesarean section. STAR Protoc. 2:100271. doi: 10.1016/j.xpro.2020.100271, 33511356 PMC7817495

[ref32] Hourigan S. K. Dominguez-Bello M. G. Mueller N. T. (2022). Can maternal-child microbial seeding interventions improve the health of infants delivered by cesarean section? Cell Host Microbe 30, 607–611. doi: 10.1016/j.chom.2022.02.014, 35550663 PMC9237654

[ref33] Hu M. Li M. Li C. Miao M. Zhang T. (2023). Effects of human Milk oligosaccharides in infant health based on gut microbiota alteration. J. Agric. Food Chem. 71, 994–1001. doi: 10.1021/acs.jafc.2c05734, 36602115

[ref34] Husso A. Pessa-Morikawa T. Koistinen V. M. Kärkkäinen O. Kwon H. N. Lahti L. . (2023). Impacts of maternal microbiota and microbial metabolites on fetal intestine, brain, and placenta. BMC Biol. 21:207. doi: 10.1186/s12915-023-01709-9, 37794486 PMC10552303

[ref35] Jakobsson H. E. Abrahamsson T. R. Jenmalm M. C. Harris K. Quince C. Jernberg C. . (2014). Decreased gut microbiota diversity, delayed Bacteroidetes colonisation and reduced Th1 responses in infants delivered by caesarean section. Gut 63, 559–566. doi: 10.1136/gutjnl-2012-303249, 23926244

[ref36] Jenmalm M. C. (2020). Re: “vaginal seeding” after a caesarean section provides benefits to newborn children: AGAINST: vaginal microbiome transfer - a medical procedure with clear risks and uncertain benefits. BJOG Int. J. Obstet. Gynaecol. 127, 906–907. doi: 10.1111/1471-0528.16176, 32133767

[ref37] Jian C. Carpén N. Helve O. de Vos W. M. Korpela K. Salonen A. (2021). Early-life gut microbiota and its connection to metabolic health in children: perspective on ecological drivers and need for quantitative approach. EBioMedicine 69:103475. doi: 10.1016/j.ebiom.2021.103475, 34256346 PMC8324810

[ref38] Johnson-Hence C. B. Gopalakrishna K. P. Bodkin D. Coffey K. E. Burr A. H. P. Rahman S. . (2023). Stability and heterogeneity in the antimicrobiota reactivity of human milk-derived immunoglobulin a. J. Exp. Med. 220:e20220839. doi: 10.1084/jem.20220839, 37462916 PMC10354535

[ref39] Jurgiel J. Gromowski T. Król J. Bomba-Opoń D. Kościółek T. Wielgoś M. (2026). The impact of maternal microbial transfer on the infant gut microbiome after cesarean delivery: a systematic review. Am. J. Obstet. Gynecol. 233, S541–S556. doi: 10.1016/j.ajog.2025.09.001, 41485840

[ref40] Kageyama S. Furuta M. Takeshita T. Ma J. Asakawa M. Yamashita Y. (2022). High-level Acquisition of Maternal Oral Bacteria in formula-fed infant Oral microbiota. MBio 13:e0345221. doi: 10.1128/mbio.03452-21, 35038919 PMC8764541

[ref41] Kaisanlahti A. Turunen J. Byts N. Samoylenko A. Bart G. Virtanen N. . (2023). Maternal microbiota communicates with the fetus through microbiota-derived extracellular vesicles. Microbiome 11:249. doi: 10.1186/s40168-023-01694-9, 37953319 PMC10642029

[ref42] Kennedy K. M. de Goffau M. C. Perez-Muñoz M. E. Arrieta M.-C. Bäckhed F. Bork P. . (2023). Questioning the fetal microbiome illustrates pitfalls of low-biomass microbial studies. Nature 613, 639–649. doi: 10.1038/s41586-022-05546-8, 36697862 PMC11333990

[ref43] Kennedy K. M. Gerlach M. J. Adam T. Heimesaat M. M. Rossi L. Surette M. G. . (2021). Fetal meconium does not have a detectable microbiota before birth. Nat. Microbiol. 6, 865–873. doi: 10.1038/s41564-021-00904-0, 33972766

[ref44] Kordy K. Gaufin T. Mwangi M. Li F. Cerini C. Lee D. J. . (2020). Contributions to human breast milk microbiome and enteromammary transfer of *Bifidobacterium breve*. PLoS One 15:e0219633. doi: 10.1371/journal.pone.0219633, 31990909 PMC6986747

[ref45] Korpela K. Helve O. Kolho K.-L. Saisto T. Skogberg K. Dikareva E. . (2020). Maternal fecal microbiota transplantation in cesarean-born infants rapidly restores Normal gut microbial development: a proof-of-concept study. Cell 183, 324–334.e5. doi: 10.1016/j.cell.2020.08.047, 33007265

[ref46] Korpela K. Salonen A. Vepsäläinen O. Suomalainen M. Kolmeder C. Varjosalo M. . (2018). Probiotic supplementation restores normal microbiota composition and function in antibiotic-treated and in caesarean-born infants. Microbiome 6:182. doi: 10.1186/s40168-018-0567-4, 30326954 PMC6192119

[ref47] Laursen M. F. Roager H. M. (2023). Human milk oligosaccharides modify the strength of priority effects in the Bifidobacterium community assembly during infancy. ISME J. 17, 2452–2457. doi: 10.1038/s41396-023-01525-7, 37816852 PMC10689826

[ref48] Lawson M. A. E. O’Neill I. J. Kujawska M. Gowrinadh Javvadi S. Wijeyesekera A. Flegg Z. . (2020). Breast milk-derived human milk oligosaccharides promote Bifidobacterium interactions within a single ecosystem. ISME J. 14, 635–648. doi: 10.1038/s41396-019-0553-2, 31740752 PMC6976680

[ref49] Li S. S. Zhu A. Benes V. Costea P. I. Hercog R. Hildebrand F. . (2016). Durable coexistence of donor and recipient strains after fecal microbiota transplantation. Science 352, 586–589. doi: 10.1126/science.aad8852, 27126044

[ref50] Lim E. S. Rodriguez C. Holtz L. R. (2018). Amniotic fluid from healthy term pregnancies does not harbor a detectable microbial community. Microbiome 6:87. doi: 10.1186/s40168-018-0475-7, 29751830 PMC5946436

[ref51] Liu W. Chen M. Duo L. Wang J. Guo S. Sun H. . (2020). Characterization of potentially probiotic lactic acid bacteria and bifidobacteria isolated from human colostrum. J. Dairy Sci. 103, 4013–4025. doi: 10.3168/jds.2019-17602, 32113772

[ref52] Liu Y. Li H.-T. Zhou S.-J. Zhou H.-H. Xiong Y. Yang J. . (2023). Effects of vaginal seeding on gut microbiota, body mass index, and allergy risks in infants born through cesarean delivery: a randomized clinical trial. Am. J. Obstet. Gynecol. MFM 5:100793. doi: 10.1016/j.ajogmf.2022.100793, 36334724

[ref53] Liu S. Luo X. Zhou L. Xie R.-H. He Y. (2024). Microbiota transplantation in restoring cesarean-related infant dysbiosis: a new frontier. Gut Microbes 16:2351503. doi: 10.1080/19490976.2024.2351503, 38748594 PMC11318963

[ref54] Lordan C. Roche A. K. Delsing D. Nauta A. Groeneveld A. MacSharry J. . (2024). Linking human milk oligosaccharide metabolism and early life gut microbiota: bifidobacteria and beyond. Microbiol. Mol. Biol. Rev. 88:e0009423. doi: 10.1128/mmbr.00094-23, 38206006 PMC10966949

[ref55] Lou Y. C. Olm M. R. Diamond S. Crits-Christoph A. Firek B. A. Baker R. . (2021). Infant gut strain persistence is associated with maternal origin, phylogeny, and traits including surface adhesion and iron acquisition. Cell Rep. Med. 2:100393. doi: 10.1016/j.xcrm.2021.100393, 34622230 PMC8484513

[ref56] Ma G. Chen Z. Li Z. Xiao X. (2024). Unveiling the neonatal gut microbiota: exploring the influence of delivery mode on early microbial colonization and intervention strategies. Arch. Gynecol. Obstet. 310, 2853–2861. doi: 10.1007/s00404-024-07843-1, 39589476

[ref57] Ma G. Peng L. Qin L. Cai R. Tan X. Gao R. (2026). Human milk microbiota: origins, determinants, and roles in maternal-infant microbial transmission and infant microbiome assembly. Front. Microbiol. 17:1790225. doi: 10.3389/fmicb.2026.1790225, 42326410 PMC13278873

[ref58] Ma G. Shi Y. Meng L. Fan H. Tang X. Luo H. . (2023). Factors affecting the early establishment of neonatal intestinal flora and its intervention measures. Front. Cell. Infect. Microbiol. 13:1295111. doi: 10.3389/fcimb.2023.1295111, 38106467 PMC10722192

[ref59] Mai V. Torrazza R. M. Ukhanova M. Wang X. Sun Y. Li N. . (2013). Distortions in development of intestinal microbiota associated with late onset sepsis in preterm infants. PLoS One 8:e52876. doi: 10.1371/journal.pone.0052876, 23341915 PMC3544792

[ref60] Matsuki T. Yahagi K. Mori H. Matsumoto H. Hara T. Tajima S. . (2016). A key genetic factor for fucosyllactose utilization affects infant gut microbiota development. Nat. Commun. 7:11939. doi: 10.1038/ncomms11939, 27340092 PMC4931012

[ref61] Mitchell C. M. Mazzoni C. Hogstrom L. Bryant A. Bergerat A. Cher A. . (2020). Delivery mode affects stability of early infant gut microbiota. Cell Rep. Med. 1:100156. doi: 10.1016/j.xcrm.2020.100156, 33377127 PMC7762768

[ref62] Moore R. L. Feehily C. Killeen S. L. Yelverton C. A. Geraghty A. A. Walsh C. J. . (2023). Ability of Bifidobacterium breve 702258 to transfer from mother to infant: the MicrobeMom randomized controlled trial. Am. J. Obstet. Gynecol. MFM 5:100994. doi: 10.1016/j.ajogmf.2023.100994, 37142190

[ref63] Morreale C. Giaroni C. Baj A. Folgori L. Barcellini L. Dhami A. . (2023). Effects of perinatal antibiotic exposure and neonatal gut microbiota. Antibiotics 12:258. doi: 10.3390/antibiotics12020258, 36830169 PMC9951864

[ref64] Mueller N. T. Differding M. K. Sun H. Wang J. Levy S. Deopujari V. . (2023). Maternal bacterial engraftment in multiple body sites of cesarean section born neonates after vaginal seeding-a randomized controlled trial. MBio 14:e0049123. doi: 10.1128/mbio.00491-23, 37074174 PMC10294643

[ref65] Mueller N. T. Hourigan S. K. Hoffmann D. E. Levy L. von Rosenvinge E. C. Chou B. . (2019). Bacterial baptism: scientific, medical, and regulatory issues raised by vaginal seeding of C-section-born babies. J. Law Med. Ethics 47, 568–578. doi: 10.1177/1073110519897732 PMC7908762 31957590

[ref66] Nolt D. O’Leary S. T. Aucott S. W. (2022). Risks of infectious diseases in newborns exposed to alternative perinatal practices. Pediatrics 149:e2021055554. doi: 10.1542/peds.2021-055554, 35104357 PMC9645715

[ref67] O’Brien C. E. Meier A. K. Cernioglo K. Mitchell R. D. Casaburi G. Frese S. A. . (2022). Early probiotic supplementation with *B. infantis* in breastfed infants leads to persistent colonization at 1 year. Pediatr. Res. 91, 627–636. doi: 10.1038/s41390-020-01350-0, 33762689 PMC8460680

[ref68] Ojima M. N. Jiang L. Arzamasov A. A. Yoshida K. Odamaki T. Xiao J. . (2022). Priority effects shape the structure of infant-type Bifidobacterium communities on human milk oligosaccharides. ISME J. 16, 2265–2279. doi: 10.1038/s41396-022-01270-3, 35768643 PMC9381805

[ref69] Pannaraj P. S. Li F. Cerini C. Bender J. M. Yang S. Rollie A. . (2017). Association between breast Milk bacterial communities and establishment and development of the infant gut microbiome. JAMA Pediatr. 171, 647–654. doi: 10.1001/jamapediatrics.2017.0378, 28492938 PMC5710346

[ref70] Rautava S. Luoto R. Salminen S. Isolauri E. (2012). Microbial contact during pregnancy, intestinal colonization and human disease. Nat. Rev. Gastroenterol. Hepatol. 9, 565–576. doi: 10.1038/nrgastro.2012.144, 22890113

[ref71] Reyman M. van Houten M. A. van Baarle D. Bosch A. A. T. M. Man W. H. Chu M. L. J. N. . (2019). Impact of delivery mode-associated gut microbiota dynamics on health in the first year of life. Nat. Commun. 10:4997. doi: 10.1038/s41467-019-13014-7, 31676793 PMC6825150

[ref72] Ricci L. Heidrich V. Punčochář M. Armanini F. Ciciani M. Nabinejad A. . (2026). Baby-to-baby strain transmission shapes the developing gut microbiome. Nature 651, 191–200. doi: 10.1038/s41586-025-09983-z, 41565819 PMC12960237

[ref73] Rodríguez J. M. (2014). The origin of human milk bacteria: is there a bacterial entero-mammary pathway during late pregnancy and lactation? Adv. Nutr. Bethesda Md 5, 779–784. doi: 10.3945/an.114.007229, 25398740 PMC4224214

[ref74] Rutayisire E. Huang K. Liu Y. Tao F. (2016). The mode of delivery affects the diversity and colonization pattern of the gut microbiota during the first year of infants’ life: a systematic review. BMC Gastroenterol. 16:86. doi: 10.1186/s12876-016-0498-0, 27475754 PMC4967522

[ref75] Sakwinska O. Foata F. Berger B. Brüssow H. Combremont S. Mercenier A. . (2017). Does the maternal vaginal microbiota play a role in seeding the microbiota of neonatal gut and nose? Benef. Microbes 8, 763–778. doi: 10.3920/BM2017.0064, 29022384

[ref76] Sela D. A. Mills D. A. (2010). Nursing our microbiota: molecular linkages between bifidobacteria and milk oligosaccharides. Trends Microbiol. 18, 298–307. doi: 10.1016/j.tim.2010.03.008, 20409714 PMC2902656

[ref77] Shao Y. Forster S. C. Tsaliki E. Vervier K. Strang A. Simpson N. . (2019). Stunted microbiota and opportunistic pathogen colonization in caesarean-section birth. Nature 574, 117–121. doi: 10.1038/s41586-019-1560-1, 31534227 PMC6894937

[ref78] Sinha T. Brushett S. Fernández-Pato A. Garmaeva S. Andreu-Sánchez S. Spreckels J. E. . (2026). Maternal influences on infant gut microbiome and health. Nature 658, 218–229. doi: 10.1038/s41586-026-10922-9, 42587158 PMC13626959

[ref79] Soto A. Martín V. Jiménez E. Mader I. Rodríguez J. M. Fernández L. (2014). Lactobacilli and Bifidobacteria in human breast Milk: influence of Antibiotherapy and other host and clinical factors. J. Pediatr. Gastroenterol. Nutr. 59, 78–88. doi: 10.1097/MPG.0000000000000347, 24590211 PMC4086764

[ref80] Sprockett D. Fukami T. Relman D. A. (2018). Role of priority effects in the early-life assembly of the gut microbiota. Nat. Rev. Gastroenterol. Hepatol. 15, 197–205. doi: 10.1038/nrgastro.2017.173, 29362469 PMC6813786

[ref81] Stewart C. J. Ajami N. J. O’Brien J. L. Hutchinson D. S. Smith D. P. Wong M. C. . (2018). Temporal development of the gut microbiome in early childhood from the TEDDY study. Nature 562, 583–588. doi: 10.1038/s41586-018-0617-x, 30356187 PMC6415775

[ref82] Tamburini S. Shen N. Wu H. C. Clemente J. C. (2016). The microbiome in early life: implications for health outcomes. Nat. Med. 22, 713–722. doi: 10.1038/nm.4142, 27387886

[ref83] Tannock G. W. (2021). Building robust assemblages of bacteria in the human gut in early life. Appl. Environ. Microbiol. 87:e0144921. doi: 10.1128/AEM.01449-21, 34469198 PMC8552902

[ref84] Valles-Colomer M. Blanco-Míguez A. Manghi P. Asnicar F. Dubois L. Golzato D. . (2023). The person-to-person transmission landscape of the gut and oral microbiomes. Nature 614, 125–135. doi: 10.1038/s41586-022-05620-1, 36653448 PMC9892008

[ref85] van Best N. Trepels-Kottek S. Savelkoul P. Orlikowsky T. Hornef M. W. Penders J. (2020). Influence of probiotic supplementation on the developing microbiota in human preterm neonates. Gut Microbes 12, 1826747–1826716. doi: 10.1080/19490976.2020.1826747, 33095113 PMC7588225

[ref86] Vuong H. E. Pronovost G. N. Williams D. W. Coley E. J. L. Siegler E. L. Qiu A. . (2020). The maternal microbiome modulates fetal neurodevelopment in mice. Nature 586, 281–286. doi: 10.1038/s41586-020-2745-3, 32968276 PMC7554197

[ref87] Wang X. Cui H. Li N. Liu B. Zhang X. Yang J. . (2024). Impact of vaginal seeding on the gut microbiome of infants born via cesarean section: a systematic review. J. Infect. 89:106348. doi: 10.1016/j.jinf.2024.106348, 39537035

[ref88] Wang W. Gu W. Schweitzer R. Koren O. Khatib S. Tseng G. . (2025). In utero human intestine contains maternally derived bacterial metabolites. Microbiome 13:116. doi: 10.1186/s40168-025-02110-0, 40329366 PMC12054239

[ref89] Wilson B. C. Butler É. M. Grigg C. P. Derraik J. G. B. Chiavaroli V. Walker N. . (2021). Oral administration of maternal vaginal microbes at birth to restore gut microbiome development in infants born by caesarean section: a pilot randomised placebo-controlled trial. EBioMedicine 69:103443. doi: 10.1016/j.ebiom.2021.103443, 34186487 PMC8254083

[ref90] Xiao L. Zhao F. (2023). Microbial transmission, colonisation and succession: from pregnancy to infancy. Gut 72, 772–786. doi: 10.1136/gutjnl-2022-328970, 36720630 PMC10086306

[ref91] Xie Z. Chen Z. Chai Y. Yao W. Ma G. (2025a). Unveiling the placental bacterial microbiota: implications for maternal and infant health. Front. Physiol. 16:1544216. doi: 10.3389/fphys.2025.1544216, 40161970 PMC11949977

[ref92] Xie Z. Chen Z. Ma G. (2025b). Dynamic changes in the pregnancy microbiome and their role in preterm birth. Front. Cell. Infect. Microbiol. 15:1683610. doi: 10.3389/fcimb.2025.1683610, 41488481 PMC12757360

[ref93] Yassour M. Jason E. Hogstrom L. J. Arthur T. D. Tripathi S. Siljander H. . (2018). Strain-level analysis of mother-to-child bacterial transmission during the first few months of life. Cell Host Microbe 24, 146–154.e4. doi: 10.1016/j.chom.2018.06.007, 30001517 PMC6091882

[ref94] Yassour M. Vatanen T. Siljander H. Hämäläinen A.-M. Härkönen T. Ryhänen S. J. . (2016). Natural history of the infant gut microbiome and impact of antibiotic treatment on bacterial strain diversity and stability. Sci. Transl. Med. 8:343ra81. doi: 10.1126/scitranslmed.aad0917, 27306663 PMC5032909

[ref95] York A. (2018). Delivery of the gut microbiome. Nat. Rev. Microbiol. 16, 520–521. doi: 10.1038/s41579-018-0059-3, 30018358

[ref96] Zellmer C. Sater M. R. A. Huntley M. H. Osman M. Olesen S. W. Ramakrishna B. (2021). Shiga toxin-producing *Escherichia coli* transmission via fecal microbiota transplant. Clin. Infect. Dis. Off. Publ. Infect. Dis. Soc. Am. 72, e876–e880. doi: 10.1093/cid/ciaa1486, 33159210

[ref97] Zhong H. Wang X.-G. Wang J. Chen Y.-J. Qin H.-L. Yang R. (2021). Impact of probiotics supplement on the gut microbiota in neonates with antibiotic exposure: an open-label single-center randomized parallel controlled study. World J. Pediatr. WJP 17, 385–393. doi: 10.1007/s12519-021-00443-y, 34331676

[ref98] Zhou L. Qiu W. Wang J. Zhao A. Zhou C. Sun T. . (2023). Effects of vaginal microbiota transfer on the neurodevelopment and microbiome of cesarean-born infants: a blinded randomized controlled trial. Cell Host Microbe 31, 1232–1247.e5. doi: 10.1016/j.chom.2023.05.022, 37327780

[ref99] Zhou P. Zhou Y. Liu B. Jin Z. Zhuang X. Dai W. . (2020). Perinatal antibiotic exposure affects the transmission between maternal and neonatal microbiota and is associated with early-onset sepsis. mSphere 5:e00984. doi: 10.1128/mSphere.00984-19 PMC7031618 32075882

[ref100] Zimmermann P. Curtis N. (2020). Effect of intrapartum antibiotics on the intestinal microbiota of infants: a systematic review. Arch. Dis. Child. Fetal Neonatal Ed. 105, 201–208. doi: 10.1136/archdischild-2018-316659, 31296695

[ref101] Zou Z.-H. Liu D. Li H.-D. Zhu D.-P. He Y. Hou T. . (2018). Prenatal and postnatal antibiotic exposure influences the gut microbiota of preterm infants in neonatal intensive care units. Ann. Clin. Microbiol. Antimicrob. 17:9. doi: 10.1186/s12941-018-0264-y, 29554907 PMC5858143

